# Development of PCR-Based Detection System for Soft Rot Pectobacteriaceae Pathogens Using Molecular Signatures

**DOI:** 10.3390/microorganisms8030358

**Published:** 2020-03-02

**Authors:** Md Niamul Kabir, Ali Taheri, C. Korsi Dumenyo

**Affiliations:** Department of Agricultural and Environmental Science, Tennessee State University, Campus Box 9543, Nashville, TN 37221, USA; mkabir@tnstate.edu (M.N.K.); ataheri1@tnstate.edu (A.T.)

**Keywords:** soft rot Enterobacteriaceae, Pectobacteriaceae, *Pectobacterium*, *Dickeya*, molecular detection, diagnostics, conserved signature protein

## Abstract

*Pectobacterium* and *Dickeya* species, usually referred to as soft rot Enterobacteriaceae, are phytopathogenic genera of bacteria that cause soft rot and blackleg diseases and are responsible for significant yield losses in many crops across the globe. Diagnosis of soft rot disease is difficult through visual disease symptoms. Pathogen detection and identification methods based on cultural and morphological identification are time-consuming and not always reliable. A polymerase chain reaction (PCR)-based detection method with the species-specific primers is fast and reliable for detecting soft rot pathogens. We have developed a specific and sensitive detection system for some species of soft rot Pectobacteriaceae pathogens in the *Pectobacterium* and *Dickeya* genera based on the use of species-specific primers to amplify unique genomic segments. The specificities of primers were verified by PCR analysis of genomic DNA from 14 strains of *Pectobacterium*, 8 strains of *Dickeya*, and 6 strains of non-soft rot bacteria. This PCR assay provides a quick, simple, powerful, and reliable method for detection of soft rot bacteria.

## 1. Introduction

Bacterial soft rot is a destructive disease in plants, notably vegetables, fruits, and ornamentals, and is caused by a group of Gram-negative bacteria collectively referred to as soft rot Enterobacteriaceae. Recently, the Proteobacterial family of Enterobacteriaceae was subdivided in to six new families, in which the soft rot pathogens were put into the new family, Pectobacteriaceae [1]. Thus, we are revising the name soft rot Enterobacteriaceae into soft rot Pectobacteriaceae (SRP). The soft rot disease occurs worldwide, mostly on fresh succulent plant tissues, and can occur in the field, in transit, in storage, and retail stores. The SRP contains two genera, *Pectobacterium* and *Dickeya,* which are globally widespread and cause high economic losses in ornamental plants, fruits, and vegetable productions [1,2,3,4,5,6,7]. Almost all vegetables are susceptible to soft rot disease, which causes a greater total loss than any other bacterial disease [2,7,8,9].

Through a series of taxonomic revisions starting in 1998, the new family Pectobacteriaceae now comprises *Brenneria, Dickeya, Lonsdalea*, *Pectobacterium, and Sodalis* genera [8,9,10,11,12]. Recently, several new *Pectobacterium* species have been reported, such as *P. polaris*, *P. peruviense, P. punjabense*, and *P. versatile*. As a result, the genus *Pectobacterium* is currently divided into 18 recognized species, including *P. carotovorum* [13,14,15,16,17,18,19,20,21,22]. Bacterial strains in the *Dickeya* genus are also divided into nine species, with the recent addition of two new species—*Dickeya lacustris* sp. nov. and *Dickeya undicola* sp. nov. [11,23,24,25,26,27,28,29].

Despite the diversity in SRP, a ubiquitous and key characteristic of SRP is their ability to produce copious amounts of plant cell wall-degrading enzymes (PCWDE), a characteristic which differentiates them from other members of the new Pectobacteriaceae or previous Enterobacteriaceae families. As a result of the devastation they cause and the research attention they have received, the SRP have been included among the top ten important plant pathogenic bacteria based on scientific or economic importance [3]. Among SRP, *P. atrosepticum*, *P. carotovorum, P. brasiliense,* and *D. dadantii* are the major pathogens causing important diseases such as aerial stem rot, blackleg, and soft rot of the potato, and many crop plants worldwide in the field and in storage [6,30,31,32,33,34,35,36,37,38]. Soft rot disease is difficult to manage, and available management strategies are not enough to reduce the effect of the disease [39]. Once the bacteria infect plants, there is no way to control this disease effectively, so accurate early pathogen detection is important for continuous SRP pathogen monitoring and prevention.

Although soft rot pathogens have received considerable research attention compared to other bacterial plant pathogens, there is still room for improvement in effective detection and diagnosis of these pathogens [4,12,40,41,42,43]. Traditional techniques of detection and identification of SRP, techniques based on isolation, bioassays, straining, microscopical observation, pathogenicity test, and biochemical methods are cumbersome [12]. These methods have two main drawbacks. First, they are used mostly to detect in vitro culturable organisms. Secondly, based on biochemical characteristics, some bacterial pathogen isolates have patterns which do not fit as a characteristic of any known genus and species. While it is relatively easy to identify bacterial soft rot disease symptomatically, separating the pathogens between the two genera can sometimes be tricky [12]. It is possible to have mixed infections of both *Pectobacterium* and *Dickeya* causing disease on a single host [12,34,44,45]. Over the last decade, we have seen the introduction of modern DNA-based approaches for the diagnosis and detection of pathogens [12,44]. Among them, PCR–based detection systems using specific primers are more reliable for detecting them. 

Various target genes, including *pmrA, pelADE, pel genes, pelY, pelI, cfa6, rhsA, recA,* and 16S rDNA, have been used until now to identify *Pectobacterium* and *Dickeya* species by PCR assays [12,46,47,48,49,50,51]. Detection systems based on these targets have suffered from one major drawback. Primers developed from these genes are mostly specific for either one or two isolates of *Pectobacterium* and *Dickeya*. As a result, these systems have challenges detecting all strains of soft rot or separating them into genera. Based on bioinformatics approaches, conserved signature proteins (CSPs) have been identified for the two major genera of SRP [45]. Among these CSPs, two are uniquely present in the *Dickeya* species, three in the *Pectobacterium* species, and two are present in both but unique to Pectobacteriaceae. Due to the conservation of these proteins, detection systems developed based on either the proteins or the genes specifying them will be specific for detecting all soft rot strains.

The main purpose of this study was to develop a PCR-based detection tool that can serve as a rapid identification technique to detect some species of SRP in the genera of *Pectobacterium* and *Dickeya*. Specifically, we have designed a set of PCR primers that can be used to detect strains of SRP as a group and also separate them into *Pectobacterium* and *Dickeya*. These tools should make easier the detection of soft rot pathogens and the distinction between the two genera.

## 2. Materials and Methods

### 2.1. Bacterial Strains, Media, and Growth Conditions

Bacterial strains used in this study and their relevant characteristics are shown in Table S1. Strains were obtained from our laboratory culture collection, including the collection of Arun Chatterjee. Bacteria were grown at 28 °C or 37 °C in Luria broth (LB), nutrient yeast agar (NY), or King’s B (KB) media. The components and preparation of all media have been previously described [52]. 

### 2.2. Genomic DNA Extraction

Genomic DNA was extracted from overnight broth cultures in LB, NY, or KB media using the Promega Wizard Genomic DNA Purification kit (Promega, Madison, WI, USA). Rehydrated and purified DNA was stored in TE buffer at −20 °C.

### 2.3. Primer Design and Optimization

Primers for this study were designed from conserved regions using the sequences of the genes for proteins listed in Table S2. These proteins have been identified previously to exist in *Pectobacterium* alone, *Dickeya* alone, or both [45,52]. Based on the above information, these CSPs were used to design primers for the detection of both *Pectobacterium* and *Dickeya* species separately and together. Primers were picked from conserved segments of CSP-coding sequences in a series of steps. First, each CSP was blasted against the NR (nonredundant) protein database at NCBI. Only CSPs whose coding sequences hit SRP, *Pectobacterium,* or *Dickeya* alone were retained and used for further development. The coding sequence of each CSP was blasted against SRP genomes, and returning sequences were aligned in a multiple sequence alignment. Primers were then hand-picked in conserved regions and analyzed for properties and compatibility with the Oligo Analyzer tool (https://www.idtdna.com/analyzer/Applications/OligoAnalyzer/). Each primer pair (Table 1) was tested by PCR using the Eppendorf Mastercycler Nexus X1. 

### 2.4. End Point PCR Conditions

Test PCR using *P. carotovorum* Ecc71 and *Dickeya dadantii* 3937 genomic DNA as the template was performed with gradient annealing temperature to determine the optimum annealing temperature for each primer pair. A 25 µL (microliter) PCR reaction contained: 1 µL (100 ng) DNA, 0.25 µL (1.25 units) Taq DNA polymerase (Thermo Fisher Scientific, Waltham, MA, USA), 2.5 µL of 10 × PCR buffer, 0.25 µL of 20 mM dNTPS, 0.25 µL each of 100 pmol each of forward and reverse primer, and 21.5 µL of sterile distilled water. Gradient PCR conditions were as follows: initial denaturation of 94 °C for 5 min (min), followed by 45 cycles at 94 °C for 1 min, 45−62 °C or 45−57 °C for 1 min, 72 °C for 1 min, and a final extension step of 72 °C for 5 min. Primers were tested with different genomic DNA listed in Table 1. Among the seven tested primer pairs, we selected the best three pairs for this study (SR1F-SR1R1 primer pair for SRP, Pcc3F-Pcc3R for *Pectobacterium,* and Dda1F-Dda1R for *Dickeya* species), and their optimized PCR protocol was run in a 15 µL reaction volume. The conditions for PCR were as follows: initial denaturation of 94 °C for 5 min, followed by 45 cycles of second denaturation 94 °C for 1 min, annealing (44.9 °C for SR1F-SR1R1, 49.9 °C for Pcc3F-Pcc3R, and 56.6 °C for Dda1F-Dda1R) for 1 min, extension 72 °C for 1 min, and a one-time final extension step of 72 °C for 5 min. The products of PCR (5 µL) were separated with 2% agarose in Tris-Boric EDTA (TBE; 0.5x) buffer and stained with ethidium bromide.

### 2.5. DNA Quantification and Plotting of Standard Curves by qPCR

Bacterial genomic DNA (gDNA) concentrations were measured using a Synergy H1 hybrid spectrophotometer microplate reader (Biotek, Winooski VT, USA). For standard curve and sensitivity testing, 100 ng µL^−1^ gDNA was 10–fold serially diluted with concentrations ranging from 100 ng µL^−1^ to 1 pg µL^−1^. These diluted samples were used as a template for quantitative real-time PCR (qPCR) using SYBR green. The *Pectobacterium*-specific primer set (Pcc3F–Pcc3R) and *Dickeya*-specific primer set (Dda1F–Dda1R) were used to run qPCR to test the sensitivity of detection with serial dilutions of Ecc71 and Dd3937 gDNA. Each dilution had three technical replications in each qPCR reaction, and every experiment was repeated two times. Standard curves were used to estimate the detection limits of each primer set and to quantify target soft rot bacterial gDNA in the samples. The detection threshold was calculated automatically using StepOne software (Applied Biosystems, Foster City, CA, USA).

The 25 µL qPCR reaction contained 11.25 µL SYBR green with real master mix (Applied Biosystems, Foster City, CA, USA), 0.125 µL each of forward and reverse primers (100 µm), 12.5 µL of sterile distilled water, and 1 µL of template gDNA. The qPCR reaction was performed with the StepOnePlus Real-Time PCR systems using MicroAmp fast optical 96-well reaction plates. The reaction conditions were as follows: initial denaturation at 94 °C for 5 min, followed by 40 cycles of denaturation at 94 °C for 1 min, annealing at 49.9 °C (for Pcc3F–Pcc3R) or 56.6 °C (for Dda1F–Dda1R) for 1 min, and extension at 72 °C for 1 min. It was considered a positive for the presence of the target DNA when reactions with threshold cycle (C_T_) values are ≤ 35.

### 2.6. Phylogenetic Analysis

The sequence of global regulatory protein RsmC (Dd586-0685 global regulatory protein) was used in a blast search against the whole NR (nonredundant) GenBank protein database. The sequences of Pectobacteriaceae (including non-soft rot genera) proteins with significant similarity were obtained from the GenBank database. These sequences were aligned with the Clustal W (Clustal Omega) program using default parameters [53], and the output file was formatted using MEGA 6 software. Phylogenetic trees for the dataset were constructed using the neighbor-joining program with MEGA 6 software [54,55]. Based on 500 resamplings, the stability of the relationships was evaluated by performing bootstrap analyses of the neighbor-joining data.

## 3. Results

### 3.1. Polymerase Chain Reaction Assay

The main purpose of this study was to develop a PCR-based system that can serve as a rapid identification technique to detect soft rot Pectobacteriaceae, *Pectobacterium* spp., and *Dickeya* spp. Twenty-eight bacterial strains (Table S1) belonging to some species of *Pectobacterium* and *Dickeya* genera, Enterobacteriaceae, and non-enterobacteria were used in this study. Of the 28, 14 were *Pectobacterium* strains, eight were *Dickeya* strains, and six were non-soft rot bacteria—of which, four were Enterobacteriaceae strains, and the remaining two were non-Enterobacteriaceae strains. Due to the recent extensive revision of taxonomy and nomenclature of this group, we were not immediately able to assign these strains to species levels based on the latest taxonomy. Therefore, we grouped the strains simply at a generic level. 

**Figure 1:**
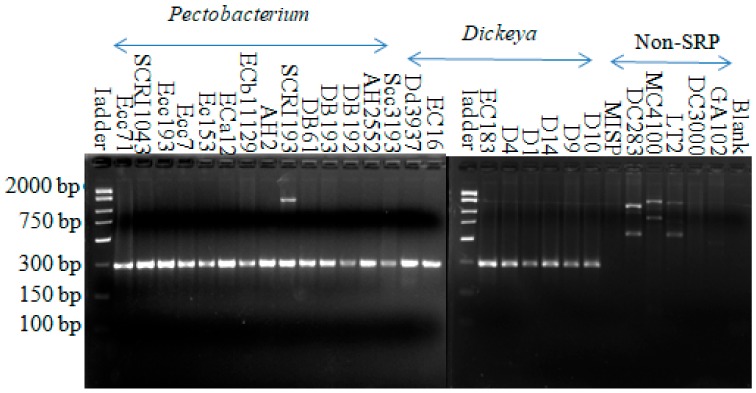
Amplification from all *Pectobacterium* and *Dickeya* strains with the soft rot-specific primer (SR1F-SR1R1). The names of the strains are indicated above each lane. The product size is around the expected value of 299 bp. Fifteen-microliter reactions were carried out, followed by 45 cycles of annealing at 44.9 °C for 1 min and an extension of 72 °C for 1 min.

When the RsmC protein (Dd586-0685 homologue) was first described as a global regulatory protein, it was also reported to be present only in soft rot bacteria using Southern blot analysis with the genomic DNA of soft rot bacteria and PCR with the *rsmC* primers on diverse strains from Pectobacteriaceae and Enterobacteriaceae [52]. These findings suggested that *rsmC* is both conserved and specific to soft rot Pectobacteriaceae. Interestingly, many years and sequenced bacterial genomes later, this protein remains essentially Pectobacteriaceae-specific. Amplification with soft rot-specific primers (SR1F-SR1R1), which were designed for the *rsmC* sequence, yielded a product with an expected size around 299 bp from all soft rot Pectobacteriaceae strains comprising both *Pectobacterium* and *Dickeya* strains. All non-SRPs which served as negative controls did not show amplification of this specific band (Figure 1).

**Figure 2:**
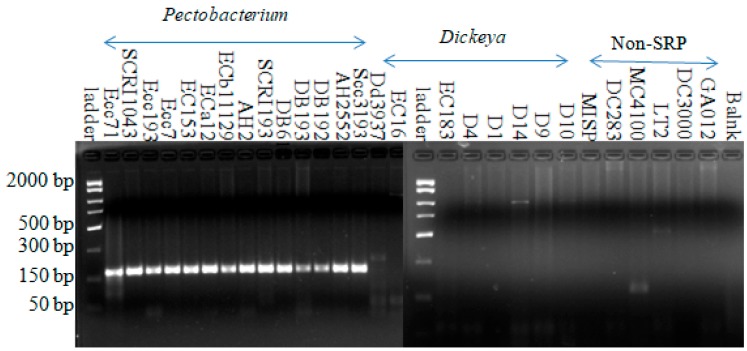
Amplification with *Pectobacterium*-specific primer (Pcc3F-Pcc3R) from all *Pectobacterium* strains yielded a product with the expected size around 177 bp. Fifteen microliter reactions were carried out in 45 cycles with annealing at 49.9 °C for 1 min and an extension at 72 °C for 1 min.

Using the *Pectobacterium*-specific primer set (Pcc3F-Pcc3R), amplification from all 14 *Pectobacterium* strains yielded a 177-bp DNA fragment in PCR. Using this primer set, all *Dickeya* species, non-soft rot Enterobacteria, and non-Enterobacterial strains did not amplify this specific fragment (Figure 2). Although faint bands were produced with *Dickeya dadantii* D14, *Escherichia coli* MC4100, and *Salmonella* LT2, none of these were in the same size range. This indicates that this primer set is specific for *Pectobacterium* species.

**Figure 3:**
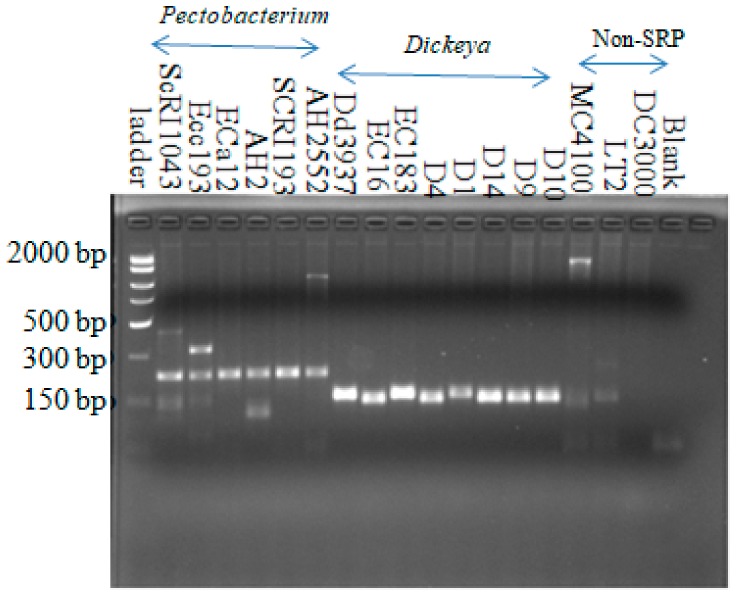
Amplification from all *Dickeya* strains with the *Dickeya*-specific primer set (Dda1F-Dda1R). The product size is around the expected size of 157 bp. Six *Pectobacterium* strains generated about 220-bp bands. Three others, two Enterobacteriaceae (MC4100 and LT2), and *Pseudomonas syringae* (DC3000) strains did not show the 157-bp bands. Fifteen-microliter reactions were carried out in 45 cycles with annealing at 56.6 °C for 1 min and an extension at 72 °C for 1 min.

All *Dickeya* strains yielded an expected 157-bp DNA fragment in PCR using the Dda1F–Dda1R primer set (Figure S1). Using this same primer set, seven out of fourteen *Pectobacterium* strains used in this study generated products in the size range of 220 bp, one of these being a very faint band on the gel. The remaining seven *Pectobacterium* strains, four non-soft rot Pectobacteriaceae, and two non-Enterobacteriaceae strains did not yield amplified fragments (Figure S1). To clearly show that it is possible to distinguish between *Pectobacterium* and *Dickeya* strains using this primer set, another PCR reaction was run with Dda1F-Dda1R primer using six *Pectobacterium* strains that produced 220-bp bright bands in the previous reaction (Figure S1). These six *Pectobacterium* strains were SCRI1043, Eca12, Ecc193, AH2, SCRI193, and AH2552. All other strains were same as in Figure S1. These six *Pectobacterium* strains yielded a product in the range size of 220-bp bands that were clearly distinguishable from the 157-bp product obtained from *Dickeya* strains (Figure 3). There were also slight differences in the sizes of the amplified products with this primer set, although the signal intensity appeared similar in all *Dickeya* strains. We speculate that there are slight differences in this locus among the *Dickeya* strains we tested. A summary of the PCR amplification results using the primers designed in this study is shown in Table 2.

### 3.2. Sensitivity of the Polymerase Chain Reaction Assay

We also used these primers in qPCR to test the sensitivity of the primers to detect these bacteria. qPCR was performed using serial dilutions of *Pectobacterium* (Ecc71) and *Dickeya* (Dd3937) gDNA ranging in concentration from 100 ng to 1 pg per reaction. *Pectobacterium*-specific (Pcc3F-Pcc3R) and *Dickeya*-specific (Dda1F-Dda1R) primer sets were used for qPCR analysis of their respective genomic DNAs. The threshold cycle (C_T_) was 24.84 cycles for gDNA (1 pg/µL) of Ecc71 by *Pectobacterium*-specific primers (Figure S2A) and 27.29 cycles for gDNA (1 pg/µL) of Dd3937 by *Dickeya*-specific primers (Figure S2B). Both Pcc3F-Pcc3R and Dda1F-Dda1R primer sets could be used to detect up to 1 pg/µL DNA. These results indicate that both primer sets can be used to specifically detect SRP pathogens with very low inoculum levels. 

### 3.3. Phylogenetic Analysis Based on Regulatory Protein, RsmC

**Figure 4:**
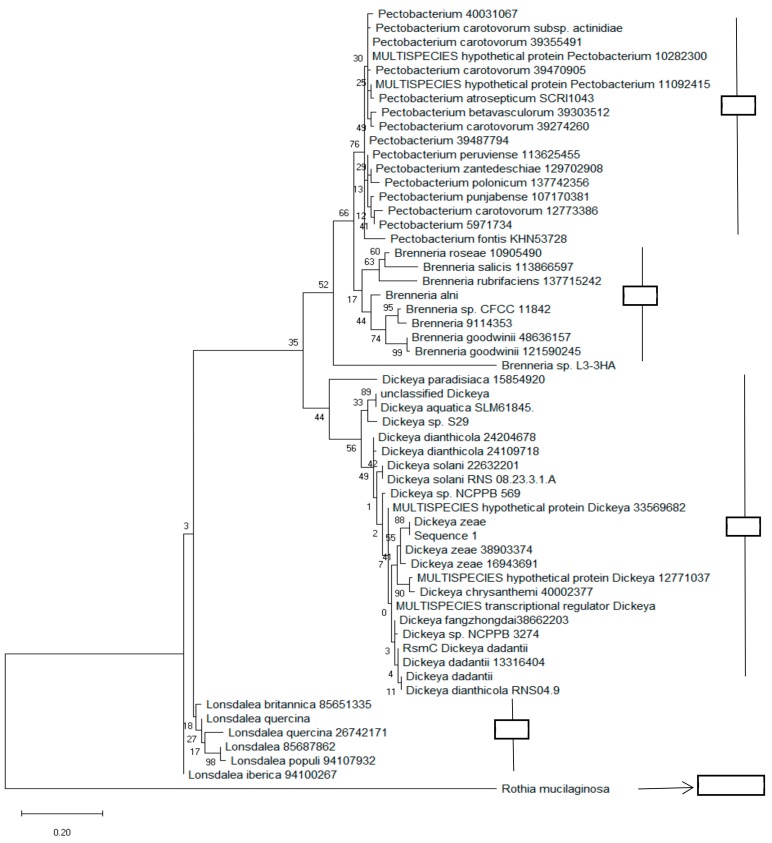
Phylogenetic tree constructed based on the *rsmC* sequence. The branching pattern was constructed using the neighbor-joining method [54]. The numbers which are present at the nodes representing the levels of bootstrap support based on a neighbor-joining study of a set of 500 resampled data. The evolutionary distances were calculated by the maximum composite likelihood method [55]. It also calculates the base substitutions per site. The whole phylogenetic tree was generated using MEGA 6 software [39].

Since *rsmC* remained essentially Pectobacteriaceae-specific and its sequence could be used to distinguish these bacteria from others, we wanted to determine if we could use it for the phylogenetic analysis of both soft rot and non-soft rot Pectobacteriaceae as well. For that, we constructed a phylogenetic tree of Pectobacteriaceae strains based on RsmC (Dd586-0685) protein sequences. The sequences were obtained from the NCBI database using a blast search against the whole NR (nonredundant) GenBank database based on the sequence of global regulatory protein RsmC (Dd586-0685). Based on the analysis, the four taxa (*Pectobacterium*, *Brenneria*, *Dickeya*, and *Lonsdalea*) were clearly divided into four clades (I -IV). Clade I groups together all the proteins from the *Pectobacterium* species, while the third clade comprises sequences from the *Dickeya* species. We also noted that there was slightly more diversity among the *Dickeya* species than among the *Pectobacterium* species (Figure 4).

## 4. Discussion

Traditional techniques, including microbiological, serological, and biochemical methods for the detection and identification of plant pathogenic bacteria, are time-consuming and, most of the time, do not have enough sensitivity and specificity [12,56]. As a result, these methods are not specifically suited for the routine analysis of many samples [56]. At times, there are also problems of low reproducibility of detection by phenotypic characteristics, lack of phylogenetic meaning, and artificial negative results due to injured bacteria [57]. As high-throughput DNA sequencing becomes common and costs reduce, pathogen detection and diagnostic techniques are slowly progressing from these traditional methods to molecular methods [58,59]. This change is expected to continue into the future as more technologies are developed [60,61]. The overall aims of all these techniques have remained the same—accurate, reliable, and fast identification and differentiation of specific bacteria from others and of the disease symptoms they cause from those by other causes. Nucleic acids-based technologies, such as conventional PCR with its variants and qPCR, have some advantages over traditional methods. Here, in vitro culture of the pathogens is not necessary for their identification [12,56]. Additionally, PCR-based methods are reliable, cost-effective, specific, sensitive, and rapid to detect pathogens from environmental samples. Detection techniques for plant pathogenic bacteria have greatly benefited from these modern technological advancements, which is needed to identify and monitor plant diseases [56]. However, these approaches have some limitations, such as difficulty in distinguishing between viable and nonviable bacterial cells [62], possible false-positives with nontargets, and a limitation of detection by the presence of inhibitors in host extracts for PCR reactions [63]. These downsides are systematically overcome with progressive improvements in the techniques. 

Three different pairs of primers were developed and used in this study, one for *Pectobacterium* spp. (Pcc3F-Pcc3R), one for *Dickeya* spp. (Dda1F-Dda1R), and the third one (SR1F-SR1R1) for soft rot bacteria comprising both *Pectobacterium* and *Dickeya* species. The *Pectobacterium*-specific primer set could detect all tested *Pectobacterium* strains, and *Dickeya*-specific primers could also detect all strains of *Dickeya* species that we tested. The global regulatory gene *rsmC*, on which the soft rot-specific primer was based, was first described in 1999 as a regulator of virulence and extracellular enzyme production in *Pectobacterium carotovorum* [52]. At the time, the authors observed through Southern blot hybridization analysis and PCR that this gene was only present in SRP. Probably because of the number of strains tested and lack of the complete genome sequence data at the time, the utility of the *rsmC* sequence as a molecular diagnostic tool was not immediately considered. It is therefore not surprising that the genomic analysis work of Naushad et al. [45] also identified *rsmC* as a soft rot-specific gene [52]. The most recent database search also returned proteins from members of Pectobacteriaceae. There were significant scores with proteins from *Serratia* spp. and *Samsonia erythrinae*, both members of the new family of Yersiniaceae. To our knowledge, this is the first primer set yet developed that can detect *Pectobacterium* and *Dickeya* species together and separately.

We showed in this study that the *rsmC* sequence could be used to separate strains of *Pectobacterium, Dickeya, Brenneria,* and *Lonsdalea* phylogenetically. Despite the divergence of *rsmC* sequences that allows it to be used for a phylogenetic analysis of the Pectobacteriaceae family, our diagnostic system could still correctly detect both soft rot genera, because the primers were specially designed for regions of the *rsmC* gene that are conserved in both genera. The primers we designed here are a first and an important step in the analysis of some *Pectobacterium* and *Dickeya* species. As our developed primers could detect several *Pectobacterium* and *Dickeya* alone and together, so this genome-based approach in developing a detection system can be a useful tool in research work to develop an effective detection system of other pathogen groups.

Each of the two genera, *Pectobacterium* and *Dickeya,* is comprised of twenty-seven species [7,8,13,64,65,66]. Some of these species in both genera are new and have only existed for a short time, following the period of heavy revision in taxonomy and phylogeny of soft rot bacteria. As a result, we were unable to test our primer set with strains belonging to all presently known species of both genera. The collection of strains belonging to all species of *Pectobacterium* and *Dickeya* across different phytosanitary jurisdictions presented a logistical hurdle that we could not overcome within the period of this project. The above shortfall notwithstanding, we anticipate that the genome-directed, PCR-based molecular diagnostic system we described here will be useful for monitoring the presence of soft rot bacteria under various conditions, whether in the field or in storage or in seed certification programs for blackleg/soft rot-free planting materials. We hope that this method will prove valuable in the routine detection of soft rot bacteria for environmental studies. We anticipate that the system will be further improved by others in an ongoing effort to improve pathogen detection and diagnosis. For example, it should be possible to develop another equally reliable detection system using the conservation of the RsmC protein sequence based on immunogenic properties.

## 5. Conclusions

In conclusion, we have developed a PCR-based detection technique which will be very helpful for detecting SRP pathogens with specificity and sensitivity. This detection method can also be valuable in developing an efficient monitoring and management system to manage soft rot disease.

## Figures and Tables

**Table 1:** List of oligonucleotide primers used for conventional PCR amplification.

Gene	Primer Name	Sequence	Annealing Temp (°C)	Product Size (bp)
Dd586_0685 global regulatory protein (RsmC)	SR1F	5′ATGAGTCTGATATTTGG 3′	44.9	299
	SR1R1	5′AGCGTMCTRADMRGMTTTTT 3′	44.9	
Dd586_2255 hypothetical protein	SR2F	5′ATGGGGCAATCAGTTGTTTT 3′	50	240
	SR2R	5′ATYACGCAAACCTCCTTTA 3′	50	
Pecwa_1592 hypothetical protein	Pcc3F	5′GGGATTCGAAAAATTACTGGCTG 3′	49.9	177
	Pcc3R	5′GCTTTTCTTTCATCAACCA 3′	49.9	
Pecwa_3132 hypothetical protein	Pcc1F	5′ GACMGRATGAATGCCAATCTGA 3′	53.1	391
	Pcc1R	5′GCGGTGAAGATAATATCGG 3′	53.1	
Pecwa_0772 hypothetical protein	Pcc2F	5′CTACTCACCTCTGCCCAAGTC 3′	60.4	112
	Pcc2R	5′CATAACCAMACGGGGMCATTGCCG 3′	60.4	
Dd586_1497 hypothetical protein	Dda1F	5′TGTTGGACGCAATACAGRGAAAG 3′	56.6	157
	Dda1R	5′TCACTCTCCATAGGTGGCATG 3′	56.6	
Dd586_0422 hypothetical protein	Dda2F	5′GCCGKAAATCCTGGGTGCGTGA 3′	62.1	245
	Dda2R	5′GGCACCCACTCCGGCGTAAAC 3′	62.1	

**Table 2:** PCR amplification with primers developed in this study.

	Primers		
	SR1F–SR1R1	Pcc3F–Pcc3R	Dda1F–Dda1R
Detected Bacteria	Both *Pectobacterium* and *Dickeya*	*Pectobacterium*	*Dickeya*
*Pectobacterium Species*			
Ecc71	+	+	-
Ecc193	+	+	-^x^
Ecc7	+	+	-^x^
EC153	+	+	-
AH2	+	+	-^x^
SCRI193	+	+	-^x^
DB61	+	+	-
DB193	+	+	-^x^
DB192	+	+	-
SCRI1043	+	+	-^x^
Eca12	+	+	-^x^
Ecb11129	+	+	-
AH2552	+	+	-
Scc3193/WPP163	+	+	-
*Dickeya* species			
Dd3937	+	-	+
Ec16	+	-	+
Ec183	+	-	+
D1	+	-	+
D4	+	-	+
D9	+	-	+
D10	+	-	+
D14	+	-^x^	+
*Erwinia tracheiphila*			
MISP	-	-	-
*Pantoea stewartii*			
DC283	-^x^	-	-
*Escherichia coli*			
MC4100	-^x^	-	-^x^
*Pseudomonas syringae*			
DC3000	-	-	-
*Agrobacterium tumefaciens*			
GA012	-	-	-
*Salmonella enterica* subsp. *enterica* serovar *Typhimurium*			
LT2	-	-	-

+ Target DNA-amplified product with expected size using the corresponding primer pair. − No amplification with corresponding primer pair. -^x^ Weak nonspecific amplification with different product size.

## Supplementary Materials

Supplementary materials can be found at https://www.mdpi.com/2076-2607/8/3/358/s1. Table S1. Bacterial Strains. Table S2. Proteins whose coding sequences were used to design primers. Figure S1. Amplification with *Dickeya* specific primer (Dda1F-Dda1R). All *Dickeya* strains yielded a product with the expected size of 157-bp. Seven *Pectobacterium* strains yielded products in the range size of 220-bp DNA fragment. Other seven *Pectobacterium* strains, two Entrobacteriaceae, two Erwiniaceae, and two non Enterobacteriales strains did not yield any fragment. Fifteen µl reactions were carried out in 45 cycles with annealing at 56.6 °C for 1 min and extension at 72 °C for 1 min. Figure S2A. Standard curves showing real-time PCR assay C_T_ values vs. template DNA concentrations from Ecc71 with Pcc3F-Pcc3R primer set. Here Y = threshold cycles (C_T_) of target DNA detected, x = amount of target DNA (pg) used as a template to generate each data point in the standard curve. Twenty five µl reactions were carried out in 40 cycles with annealing at 49.9 °C for 1 min and extension at 72 °C for 1 min. Red color represents target samples and green color represents positive control. The R^2^ for the plot is 0.89. Figure S2B. Standard curves showing real-time PCR assays. C_T_ values vs template DNA concentrations from Dd3937 with Dda1F-Dda1R primer set. Here Y-axis represents threshold cycles (C_T_) of target DNA detected and the, X-axis represents amount of target DNA (pg) used as a template to generate each data point in the standard curve. Twenty-five µl reactions were carried out in 40 cycles with annealing at 56.6 °C for 1 min and extension at 72 °C for 1 min. Blue color represents positive control and red color represents positive control. The R^2^ for the plot is 0.99.
