# Supplementary material for: Development of PCR-Based Detection System for Soft Rot Pectobacteriaceae Pathogens Using Molecular Signatures
Source: Microorganisms. 2020 Mar 2;8(3):358. doi: 10.3390/microorganisms8030358 (PMC7143467; doi:10.3390/microorganisms8030358)
Supplement: Supplementary file 1 [file microorganisms-08-00358-s001.pdf]

Supplemental materials:

Table S1: Bacterial Strains

*Bacterial Strains	Relevant Characteristics	Reference or Source
Pectobacterium species		
Ecc71	Wild Type	[1]
Ecc193	Wild Type	[2]
Eca12	Wild Type	[2]
SCRI193	Wild Type	[2]
Ecc7	Wild Type	Lab collection
EC153	Wild Type	[3]
DB61	Wild Type	Lab collection
DB193	Wild Type	Lab collection
DB192	Wild Type	Lab collection
AH2	Wild Type	[4]
SCRI1043	Wild Type	Lab collection
Ecb11129	Wild Type	Lab collection
AH2552	Wild Type	[4]
Scc3193	Wild Type	[5]
Dickeya Species		
Dd3937	Wild Type	[6]
Ec16	Wild Type	[7]
Ec183	Wild Type	Lab collection
D1	Wild Type	Lab collection
D4	Wild Type	Lab collection
D9	Wild Type	Lab collection
D10	Wild Type	Lab collection
D14	Wild Type	Lab collection
Erwinia tracheiphila		
MISpSp	Wild Type	[8]
Escherichia coli		
MC4100	Wild Type	Lab collection
Pseudomonas syringae pv tomato		
DC3000	Wild Type	[9]
Pantoea stewartii		
DC283	Wild Type	[10]
Agrobacterium tumefaciens		
GA012	Wild Type	Lab collection
Salmonella enterica subsp. *enterica* serovar *Typhimurium*		

LT2	Wild Type	Lab collection

*Because of continuing revision in the taxonomy and nomenclature of soft rot bacteria, we have decided not to assign species to the strains. Thus, we are grouping all *Pectobacterium* strains together and all *Dickeya* strains together.

Table S2: Proteins whose coding sequences were used to design primers

Source Genome	*Protein	Accession	Genus detected
Dickeya dadantii Ech586	Dd586_0685 global regulatory protein	YP_003332283.1	*Dickeya* and *Pectobacterium*
Pectobacterium wasabiae WPP163	Pecwa_1592 hypothetical protein	YP_003258989.1	*Pectobacterium*
Pectobacterium wasabiae WPP163	Pecwa_3132 hypothetical protein	YP_003260481.1	*Pectobacterium*
Pectobacterium wasabiae WPP163	Pecwa_0772 hypothetical protein	YP_003258198.1	*Pectobacterium*
Dickeya dadantii Ech586	Dd586_1497 hypothetical protein	YP_003333070.1	*Dickeya*
Dickeya dadantii Ech586	Dd586_0422 hypothetical protein	YP_003332023.1	*Dickeya*

*Identified by Naushad HS, Lee B, and Gupta RS [11].

Figure S1: Amplification with *Dickeya* specific primer (Dda1F-Dda1R). All *Dickeya* strains yielded a product with the expected size of 157-bp. Seven *Pectobacterium* strains yielded products in the range size of 220-bp DNA fragment. Other seven *Pectobacterium* strains, two Entrobacteriaceae, two Erwiniaceae and two non Enterobacteriales strains did not yield any fragment. Fifteen µl reactions were carried out in 45 cycles with annealing at 56.6 °C for 1 min and extension at 72 °C for 1 min.

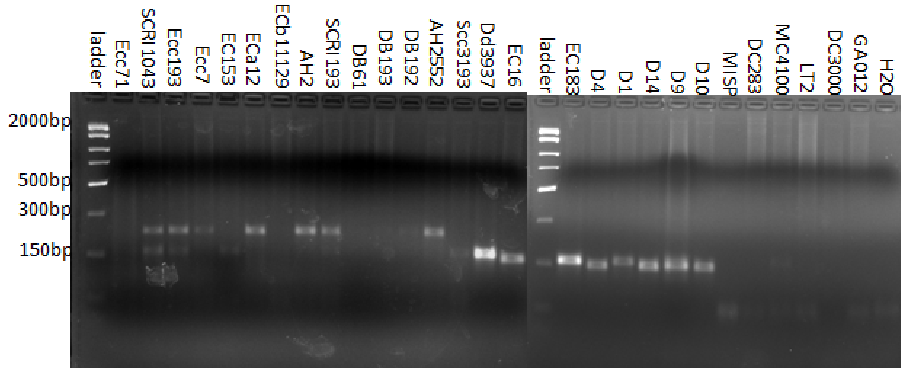

Figure S2A: Standard curves showing real-time PCR assay C_T values vs. template DNA concentrations from Ecc71 with Pcc3F-Pcc3R primer set. Here Y = threshold cycles (C_T) of target DNA detected, x = amount of target DNA (pg) used as a template to generate each data point in the standard curve. Twenty-five µl reactions were carried out in 40 cycles with annealing at 49.9 °C for 1 min and extension at 72 °C for 1 min. Red color represents target samples and green color represents positive control. The R^2 for the plot is 0.89.

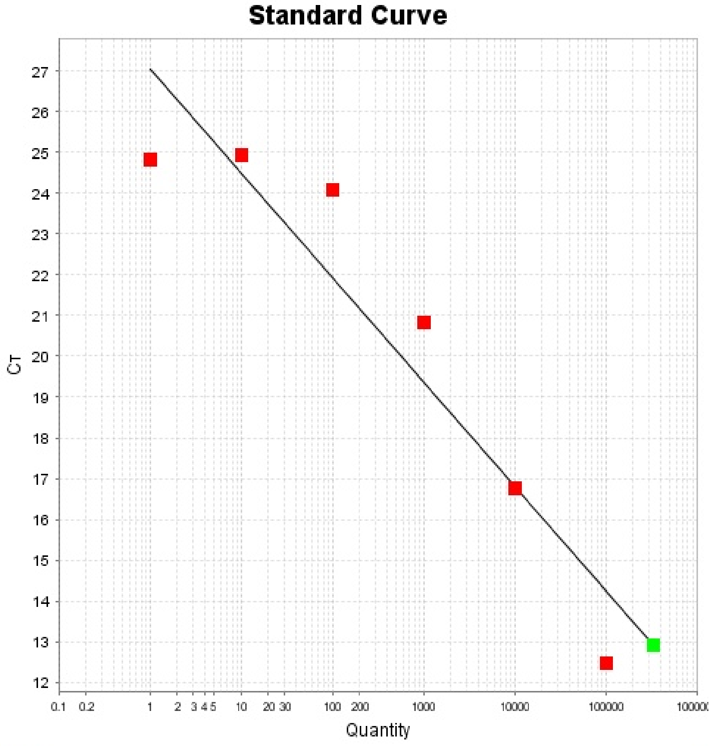

gDNA 1pg to 100ng

Figure S2B: Standard curves showing real-time PCR assays. C_T values vs template DNA concentrations from Dd3937 with Dda1F-Dda1R primer set. Here Y-axis represents threshold cycles (C_T) of target DNA detected and the, X-axis represents amount of target DNA (pg) used as a template to generate each data point in the standard curve. Twenty-five µl reactions were carried out in 40 cycles with annealing at 56.6 °C for 1 min and extension at 72 °C for 1 min. Blue color represents positive control and red color represents positive control. The R^2 for the plot is 0.99.

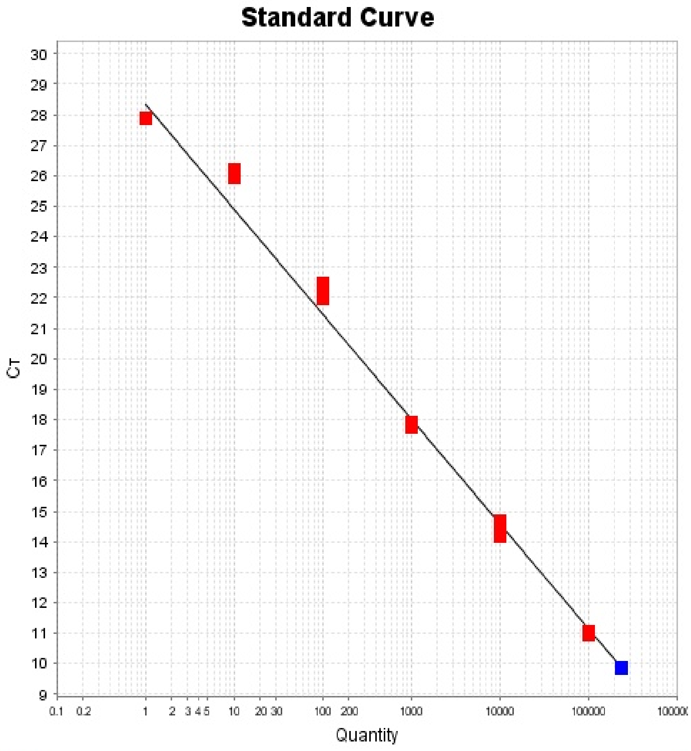

gDNA 1pg to 100ng

References

Commented [KD1]: These were previously in the main document. We decided to separate them from those of the main manuscript. Accordingly, the number of refences in the main manuscript has reduced.

1. Zink, R.T.; Kemble, R.J.; Chatterjee, A.K. Transposon Tn5 mutagenesis in *Erwinia carotovora subsp. carotovora* and *Erwinia carotovora subsp. atroseptica. J. Bacteriol*. **1984**, *157*, 809–814.
2. Salmond, G.P.C.; Hinton, J.C.D.; Gill, D.R.; Perombelon, M.C.M. Transposon mutagenesis of *Erwinia* using phage *λ* vectors. Mol. *Gen. Genet*. **1986**, *203*, 524–528.
3. Kraght, A.J.; Starr, M.P. Fermentation of galacturonic acid and glucose by a strain of *Erwinia carotovora*. *J. Bacteriol*. **1952**, *64*, 259–264.
4. Frederick, R.D.; Chiu, J.; Bennetzen, J.L.; Handa, A.K. Identification of a pathogenicity locus, *rpfA*, in *Erwinia carotovora* subsp. *carotovora* that encodes a two-component sensor-regulator protein. *Mol. Plant-Microbe Interact*. **1997**, *10*, 407–415.
5. Pirhonen, M.; Flego, D.; Heikinheimo, R.; Palva, E.T. A small diffusible signal molecule is responsible for the global control of virulence and exoenzyme production in the plant pathogen *Erwinia carotovora*. *EMBO J*. **1993**, *12*, 2467–2476.
6. Kotoujansky, A.; Diolez, A.; Boccara, M.; Bertheau, Y.; Andro, T.; Coleno, A.; van der Wolf, J.M.; Kastelein, P.; Van Beckhoven, J.R.C.M.; Van Den Brink, M. Molecular cloning of *Erwinia chrysanthemi* pectinase and cellulase structural genes. *EMBO J*. **1985,** *4*, 781–785.
7. Chatterjee, A.K.; Buchanan, G.E.; Behrens, M.K.; Starr, M.P. Synthesis and excretion of polygalacturonic acid trans- eliminase in *Erwinia*, *Yesinia*, and *Klebsiella* species. *Can. J. Microbiol.* **1979,** *25*, 94–102.
8. Rojas, E.S.; Dixon, P.M.; Batzer, J.C.; Gleason, M.L. Genetic and virulence variability among *Erwinia tracheiphila* strains recovered from different cucurbit hosts. *Phytopathol*. **2013**, *103*, 900–905, doi:10.1094/PHYTO-11-12-0301-R.
9. Xin, X.F.; He, Y.H. *Pseudomonas syringae* pv. tomato DC3000: A model pathogen for probing disease susceptibility and hormone signaling in plants. *Annu. Rev. Phytopathol*. **2013**, *51*, 473–498.
10. Dolph, P.J.; Majerczak, D.R.; Coplin, D.L. Characterization of a gene cluster for exopolysaccharide biosynthesis and virulence in *Erwinia stewartii. J. Bacteriol*. **1988**, *170*, 865–871.
11. Naushad, H.S.; Lee, B.; Gupta, R.S. Conserved signature indels and signature proteins as novel tools for understanding microbial phylogeny and systematics: Identification of molecular signatures that are specific for the phytopathogenic genera *Dickeya*, *Pectobacterium* and *Brenneria*. *Int. J. Syst. Evol. Microbiol*. **2014**, *64*, 366–383, doi:10.1099/ijs.0.054213-0.
